# Influence of Body Mass Index on Inflammatory Profile at Admission in Critically Ill Septic Patients

**DOI:** 10.1155/2015/734857

**Published:** 2015-05-10

**Authors:** Fernando G. Zampieri, Vanessa Jacob, Hermes V. Barbeiro, Fabiano Pinheiro da Silva, Heraldo P. de Souza

**Affiliations:** ^1^Medical Investigation Laboratory 51 (LIM-51), Emergency Medicine Discipline, Faculty of Medicine, University of São Paulo, 01246903 São Paulo, SP, Brazil; ^2^Intensive Care Unit, Emergency Medicine Discipline, Hospital das Clínicas, University of São Paulo, 05403-010 São Paulo, SP, Brazil; ^3^Intensive Care Unit, Hospital Alemão Oswaldo Cruz, 01323-020 São Paulo, SP, Brazil

## Abstract

*Introduction*. Inflammation is ubiquitous during sepsis and may be influenced by body mass index (BMI). We sought to evaluate if BMI was associated with serum levels of several cytokines measured at intensive care unit admission due to sepsis. *Methods*. 33 septic patients were included. An array of thirty-two cytokines and chemokines was measured using Milliplex technology. We assessed the association between cytokine levels and BMI by generalized additive model that also included illness severity (measured by SAPS 3 score); one model was built for each cytokine measured. *Results*. We found that levels of epidermal growth factor, vascular endothelial growth factor, and interleukins 4, 5, and 13 were associated with BMI in a complex, nonlinear way, independently of illness severity. Higher BMI was associated with higher levels of anti-inflammatory interleukins. *Conclusion*. BMI may influence host response to infection during critical illness. Larger studies should confirm these findings.

## 1. Introduction

Sepsis is a major cause of mortality and morbidity and its incidence is increasing [1]. Despite the major social impact of sepsis, its physiopathology is still incompletely understood [2, 3]. Inflammation is ubiquitous during the course of critically ill septic patients and may be modulated by several patient's particularities, such as age, gender, and comorbidities [3–5]. Nevertheless, the extend by which each patient characteristic impacts the inflammatory response is unclear.

Body mass index is an important prognostic factor in general population and is associated with inflammation [6, 7]. BMI may also impact the prognosis of critically ill patients. For example, it has been suggested that obesity might be associated with improved prognosis in critically ill patients (the so-called “obesity paradox”), although reasons for this are unclear [8, 9]. It has been hypothesized that changes in BMI may alter the host response to the pathogen and inflammatory response, thereby influencing the course of critical illness and therefore prognosis, but evidence available is conflicting [9]. On the other hand, underweight patients may also display higher inflammation on specific clinical conditions [10], suggesting that the interplay between BMI and inflammation may be complex and nonlinear.

We therefore performed an explanatory analysis to evaluate the association between BMI and serum levels of several cytokines measured at ICU admission. Our initial hypothesis was that BMI would be associated with serum levels of several inflammatory markers. The subset of septic patients of a unicentric cohort of critically ill patients was used for this analysis.

## 2. Methods

This study is a subset analysis of a previous study that evaluated the association between components of acid-base status and inflammation in critically ill patients [11]. The study was approved by local ethical committee and is part of a larger project that aims to evaluate inflammation in critically ill patients (institutional approval number 1207/99).

Methods, including sample time and cytokine analysis, have been described elsewhere [11]. In brief, blood sample for cytokine analysis was collected in the morning following ICU admission. The 87 patients included in the main original study were consecutively admitted patients during the study period that fulfilled inclusion criteria and that were admitted from Sunday afternoon until early Friday morning, as it was technically not possible to process blood samples during weekends. Cytokine analysis was performed using Milliplex technology (Merck, Genese Diagnostics, Darmstadt, Germany). Illness severity was assessed through Simplified Acute Physiology Score 3 (SAPS3 score [12]). The following cytokines were included in this analysis: epidermal growth factor (EGF); vascular endothelial growth factor (VEGF); eotaxin; fibroblast growth factor 2 (FGF2); granulocyte macrophage colony-stimulating factor (GMCSF); granulocyte colony-stimulating factor (GCSF); fractalkine; interferon alpha and gamma (IFN*α* and IFN*γ*); interleukin (IL) 1*α* (IL1*α*); IL1*β*; IL1 receptor antagonist (IL1RA); IL2; IL3; IL4; IL5; IL6; IL7; IL8; IL9; IL10; IL12p40; and IL12p70; IL13; IL15; IL17; monocyte chemoattractant proteins (MCP) 1 and 3 (MCP1 and MCP3); macrophage inflammatory protein (MIP) 1*α* (MIP1*α*) and macrophage inflammatory protein 1*β* (MIP1*β*); tumor necrosis factor (TNF) *α* (TNF*α*) and tumor necrosis factor *β* (TNF*β*). C-reactive protein (CRP) levels were also evaluated as a global marker of inflammation.

In order to evaluate the association and impact of a body mass index, a generalized additive model (GAM) was created to evaluate the association of each cytokine level with BMI. SAPS3 score was added to the model in order to account for multiple possible confounders, such as illness severity and age. The main advantage of GAM is that no inference is made* a priori* regarding the type of link between predictors and outcome variable. Therefore, nonlinear association between variables and predictors can be evaluated through GAM. GAM output provides *P* value for the association between each predictor and the variable of interest, as well as a graphical plot of variable values versus smooth terms and degrees of freedom. Both SAPS3 and BMI were added as smooth terms on GAM models. The smoothing parameter estimating method was GCV (generalized cross validation) criterion. Smoothing parameters are automatically selected in order to obtain the smallest GCV possible. One model was built for each cytokine investigated. When an association between cytokine and BMI was found, we further assessed the effects of adding other relevant variables to the model, specifically, gender, pulmonary source of infection, and diabetes.

## 3. Results

**Figure 1:**
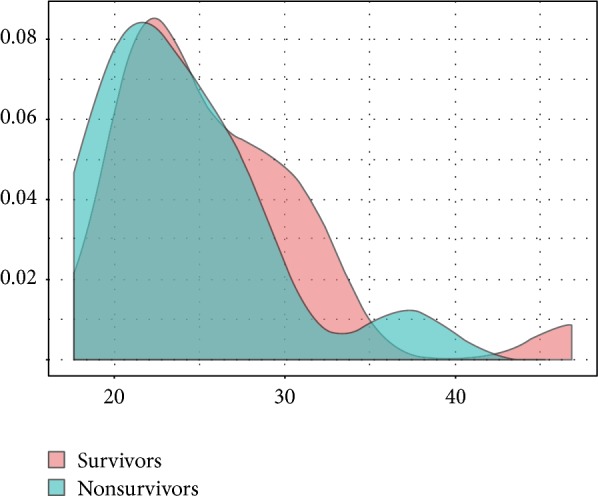
Density plot for BMI in survivors and nonsurvivors. There was no difference between groups (*P* = 0.256).

From the original 87 patients included in the main study, 41 patients were admitted due to sepsis. Thirty-three of those patients had weight measured and were therefore elected for analysis. We considered only weight values that were collected in the ward and/or emergency department before ICU admission or a body weight measured at ICU admission using a weighting machine. Patients with estimated body weight were not included. General features of the population are shown in Table 1. Mean age was 50.6 years (SD 19.6) and 60% were males. Mean body mass index was 25.2 (SD 5.8). BMI was similar in hospital survivors and nonsurvivors (26.1 ± 6.2 versus 23.8 ± 5.1; *P* = 0.256). Kernel density plot for BMI stratified by hospital discharge status is shown in Figure 1. The most common sources of infection were lungs (33%), bloodstream (9%), and soft tissue (12%). Mean SAPS3 score was 60 (IQ range 48–70). Most patients (73%) required vasopressors and close to half (42%) had acute kidney injury. Other features are shown in Table 1.

**Figure 2:**
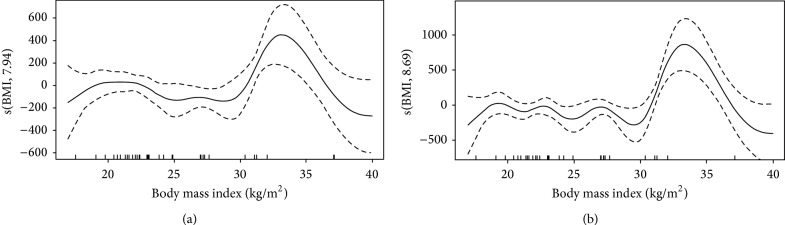
Association between EGF (a) and VEGF (b) and body mass index. Intervals are Bayesian credible intervals.

**Figure 3:**
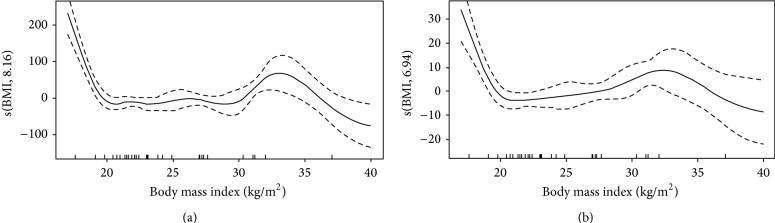
Association between IL4 (a) and IL5 (b) levels and body mass index. Intervals are Bayesian credible intervals.

**Figure 4:**
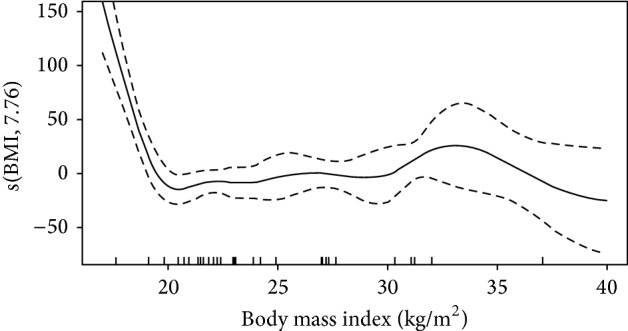
Association between IL13 levels and body mass index. Intervals are Bayesian credible intervals.

Cytokine levels and results for GAM analysis are shown in Table 2 and in Figures 2, 3, and 4. As shown in Table 2, EGF, VEGF, IL4, IL5, and IL13 were associated with BMI. Illness severity, as assessed by SAPS3 score, was associated with GCSF, IL1RA, IL6, IL8, IL15, MCP1, and CRP levels. The plots for BMI versus the smoothed coefficient for its association with cytokine levels are shown in Figures 2–4. The *y*-axis should be interpreted as how changes in BMI affect the mean value of the *y*-axis (i.e, cytokine value), with positive values meaning an increase and negative values a decrease. In brief, BMI over 30 kg/m^2^ was associated with higher EGF and VEGF levels (Figure 2). For EGF, the lower values were found for BMI between 25 and 30 kg/m^2^ (overweight patients). The association between BMI and IL4, IL5, and IL13 levels had a similar pattern (Figures 3 and 4), with higher interleukin values for low (<20 kg/m^2^) and high (above 30 kg/m^2^) BMI values, with a plateau between 20 and 30 kg/m^2^.

Additional models controlling for gender, pulmonary source of infection, and diabetes were built for EGF, VEGF, IL4, IL5, and IL13. Results are shown in Table 3. In brief, the effects of BMI remained even when other variables were added to the model (plots not shown). VEGF values were higher in female gender and when the lungs were the infection source. Lower IL13 values were found in diabetic patients (Table 3).

## 4. Discussion

In this subgroup analysis, we found that EGF, VEGF, IL4, IL5, and IL13 are related to body mass index in a nonlinear way. There was a sharp increase for EGF and VEGF levels in obese patients. For interleukin values, the association followed a U shape distribution, with lower values found in the range of patients with normal weight and overweight.

The association between VEGF levels and sepsis has been previously reported [13]. We obtained high VEGF values, similar to other reports of VEGF levels in patients with septic shock [13]. VEGF may play an important role in both endothelial dysfunction and repair in sepsis [14]. Since endothelial dysfunction appears to be involved in the pathogenesis of organ failure [15], higher VEGF levels may be necessary to repair the endothelium after an infectious insult [14]. Our data suggests that even when illness severity is taken into account, VEGF levels tend to be higher in obese patients than in nonobese individuals, which may reflect a higher previous value in this population, as reported in [16], or an increase in the stimulus to endothelial repair in obese patients.

IL4 and IL13 are anti-inflammatory cytokine that increase in sepsis and may play important roles in immune regulation [17–19]. IL13 blockade in experimental sepsis has been shown to decrease survival and increase the expression of inflammatory cytokines [20]. Blanco-Quirós et al. suggested that in septic children lower IL13 values were associated with worst prognosis, although no association between the levels of other cytokines and IL13 was found [21]. The presence of higher values of IL4 and IL13 in obese patients may suggest that obesity is associated with a more anti-inflammatory cytokine expression during critical illness.

The association between BMI and cytokine levels was largely unaltered when gender, presence for diabetes, and pulmonary source of infection were added to the model (Table 3), suggesting that the findings are robust even when other variables are accounted for in the statistical model. The association between female gender and higher VEGF values has been suggested in animal models and may be mediated by estrogen [22, 23]. The association between pulmonary source of infection and higher VEGF values has been suggested in other scenarios, such as exacerbations of cystic fibrosis [24], hypoxia, and hantavirus infections [25]. The interaction between diabetes and IL13 levels is complex and deserves further specific studies [26].

There are several constrains in our analysis. First, and more importantly, we used a limited number of patients which limits our conclusions and the external validity of the analysis. Only six patients had a BMI above 30 kg/m^2^, which may limit our conclusions regarding higher BMI values. Second, we only evaluated cytokine levels at admission and we are therefore unable to evaluate if BMI would result in a different inflammation profile over time; additionally, time elapsed between ICU admission and blood sample was variable since blood was drawn in the morning after ICU arrival. Third, we have no data regarding the previous evolution of the patient before ICU precise elapsed time. Fourth, obesity may be associated with higher background values of some inflammatory mediators which could affect the results [27]. Finally, we are unable to address if changes in immune function associated with BMI have prognostic significance in critically ill patients.

## 5. Conclusion

BMI might influence inflammatory profile of critically ill patients admitted due to sepsis. This funding should be properly evaluated in larger series of critically ill individuals.

## Figures and Tables

**Table 1:** Demographic and clinical features.

Number of patients	33
Age, years, mean ± SD	50.6 ± 19.6
Male sex, *n* (%)	20 (60)
Body mass index, kg/m^2^, mean ± SD	25.2 ± 5.8
Source of infection	
Respiratory	11 (33)
Bloodstream/intravascular catheter	9 (27)
Soft tissue/fasciitis	4 (12)
Abdominal	3 (11)
Meningitis	2 (6)
Urinary tract	1 (3)
Other	3 (11)
Illness severity	
SAPS 3 score, points, median [IQ]	60 [48–70]
SOFA score, points, median [IQ]	7 [5–9]
Vasopressor use, *n* (%)	17 (73)
Mechanical ventilation, *n* (%)	9 (27)
Acute kidney injury, *n* (%)	14 (42)
Comorbidities	
Hypertension, *n* (%)	15 (45)
Diabetes, *n* (%)	11 (33)
Chronic kidney disease, *n* (%)	8 (24)
Neoplasia, *n* (%)	1 (3)
Cirrhosis, *n* (%)	3 (10)
ICU mortality, *n* (%)	10 (30)
Hospital mortality, *n* (%)	13 (40)

**Table 2:** Serum levels and results for generalized additive model including SAPS 3 score and cytokine for the 33 included patients. ^∗^CRP levels are shown in mg/dL.

Cytokine	Serum levels pg/mL^∗^ median [IQ]	SAPS 3 score *P* value	Body mass index *P* value
EGF	153 [106; 290]	0.458	0.001
VEGF	263 [153; 429]	0.197	<0.001
Eotaxin	103 [79.23; 145]	0.200	0.159
FGF2	121 [88.62; 153]	0.133	0.100
Fractalkine	255 [147; 304]	0.711	0.776
GCSF	253 [108; 591]	<0.001	0.111
GMCSF	47.45 [27.35; 66.68]	0.577	0.625
IFN*α*	80.74 [58.01; 99.33]	0.716	0.072
IFN*γ*	16.52 [7.37; 32.57]	0.957	0.369
IL1*α*	50.42 [22.01; 153]	0.453	0.404
IL1*β*	3.60 [1.49; 6.67]	0.119	0.408
IL1RA	38.52 [17.33; 65.49]	0.013	0.191
IL2	10.03 [7.17; 14.82]	0.567	0.945
IL3	1.64 [1.06; 2.92]	0.179	0.494
IL4	18.11 [4.70; 33.67]	0.566	<0.001
IL5	3.92 [2.25; 9.44]	0.222	<0.001
IL6	70.48 [45.76; 393]	<0.001	0.188
IL7	17.93 [12.95; 30.73]	0.212	0.827
IL8	70.7 [30.49; 132]	0.012	0.087
IL9	3.32 [1.74; 5.23]	0.428	0.080
IL10	53.74 [25.11; 101]	0.471	0.916
IL12p40	46.85 [26.63; 104]	0.150	0.126
IL12p70	10.63 [6.08; 22.58]	0.680	0.879
IL13	4.22 [1.64; 13.71]	0.649	<0.001
IL15	10.62 [7.39; 18.07]	0.020	0.368
IL17	8.65 [3.2; 11.24]	0.512	0.971
MCP1	874 [325; 2101]	0.017	0.311
MCP3	42.68 [24.36; 69.91]	0.559	0.275
MIP1*α*	25.24 [18.14; 49.78]	0.463	0.728
MIP1*β*	75.14 [48.11; 97.04]	0.715	0.533
TNF*α*	34.15 [26.96; 86.96]	0.759	0.714
TNF*β*	3.85 [2.78; 8.62]	0.905	0.544
CRP	83.5 [9.91; 216.2]	0.031	0.954

**Table 3:** Results for the additional model built for EGF, VEGF, IL4, IL5, and IL13. This model included SAPS 3 score, BMI, gender, pulmonary source, and presence of diabetes. Estimates (SE) values are as follows: ^∗^163.75 (67.80); ^†^150.95 (69.41); ^‡^−20.23 (7.00).

Cytokine	SAPS 3 score *P* value	Body mass index *P* values	Female gender *P* value	Presence of diabetes *P* value	Pulmonary source *P* value
EGF	0.386	0.020	0.620	0.253	0.066
VEGF	0.573	<0.001	0.022^∗^	0.120	0.038^†^
IL4	0.673	<0.001	0.314	0.056	0.782
IL5	0.277	<0.001	0.989	0.271	0.127
IL13	0.597	<0.001	0.913	<0.001^‡^	0.122

## Abbreviations

BMI: Body mass index
EGF: Epidermal growth factor
FGF2: Fibroblast growth factor 2
GAM: Generalized additive model
GCV: Generalized cross validation
GCSF: Granulocyte colony-stimulating factor
GMCSF: Granulocyte macrophage colony-stimulating factor
IFN: Interferon
IL: Interleukin
IL1RA: IL1 receptor antagonist (IL1RA)
MCP: Monocyte chemoattractant protein
MIP: Macrophage inflammatory protein
SAPS3: Simplified acute physiology state score 3
TNF: Tumor necrosis factor
VEGF: Vascular endothelial growth factor.
